# Supplementary material for: Cross-sectional study on the prevalence of influenza and pneumococcal vaccination and its association with health conditions and risk factors among hospitalized multimorbid older patients
Source: PLoS One. 2021 Nov 16;16(11):e0260112. doi: 10.1371/journal.pone.0260112 (PMC8594840; doi:10.1371/journal.pone.0260112)
Supplement: S1 Table — (DOCX) [file pone.0260112.s001.docx]

|  | **Unadju-sted PR** | **95 % CI** | **p-value**‡ | **Unadju-sted PR** | **95% CI** | **p-value**‡ |
| --- | --- | --- | --- | --- | --- | --- |
| **Influenza Vaccination Pneumococcal Vaccination** | | | | | | |
| **Clinical risk groups** |  |  |  |  |  |  |
| Chronic heart disease | 1.03 | 0.99-1.10 | 0.15 | 0.81 | 0.67-0.98 | 0.028 |
| Chronic respiratory disease | 1.18 | 1.07-1.31 | 0.001 | 2.03 | 1.12-3.44 | 0.009 |
| Chronic liver disease | 0.89 | 0.74-1.08 | 0.25 | 0.95 | 0.58-1.56 | 0.84 |
| Chronic kidney disease | 1.17 | 1.10-1.24 | <0.001 | 1.12 | 0.87-1.44 | 0.38 |
| Diabetes mellitus | 1.04 | 1.03-1.04 | <0.001 | 1.22 | 1.12-1.33 | <0.001 |
| Rheumatic disease | 1.02 | 0.96-1.08 | 0.59 | 1.20 | 1.00-1.44 | 0.046 |
| Any malignancy † | 1.02 | 0.98-1.05 | 0.32 | 1.13 | 0.86-1.49 | 0.39 |
| Immunosuppression | 0.97 | 0.84-1.13 | 0.72 | 1.36 | 1.13-1.64 | 0.001 |
| **Health care contacts*** |  |  |  |  |  |  |
| GP visits, n 0 | *Reference* | |  | *Reference* | |  |
| 1-2 | 1.10 | 0.87-1.40 | 0.13 | 2.36 | 1.59-3.52 | <0.001 |
| 3-4 | 1.12 | 0.93-1.35 |  | 2.89 | 2.70-3.10 |  |
| ≥ 5 | 1.24 | 0.92-1.66 |  | 3.51 | 2.98-4.14 |  |
| Other outpatient physician or ED visits, n 0 | *Reference* | |  | *Reference* | |  |
| 1-2 | 1.12 | 1.01-1.23 | 0.11 | 1.02 | 0.60-1.71 | 0.62 |
| ≥ 3 | 1.09 | 0.98-1.22 |  | 1.29 | 0.46-3.62 |  |
| Hospitalizations, n 0 | *Reference* | |  | *Reference* | |  |
| 1 | 1.04 | 0.99-1.09 | 0.38 | 0.92 | 0.85-1.01 | 0.75 |
| ≥ 2 | 0.97 | 0.92-1.03 |  | 1.03 | 0.85-1.25 |  |
| Nursing home resident | 1.06 | 0.96-1.17 | 0.28 | 1.02 | 0.71-1.47 | 0.91 |
| Any home nursing visits | 1.03 | 0.96-1.11 | 0.35 | 1.15 | 0.94-1.42 | 0.18 |
| Receipt of informal care†† | 1.13 | 1.05-1.21 | <0.001 | 1.40 | 0.83-2.36 | 0.20 |
| **Health scores** |  |  |  |  |  |  |
| EQ-5D < mean§§ | 1.02 | 0.99-1.06 | 0.21 | 1.26 | 0.71-2.23 | 0.43 |
| CCI ≥ 7** | 1.13 | 1.06-1.21 | <0.001 | 1.07 | 0.86-1.34 | 0.52 |

Abbreviations: CCI, Charlson comorbidity index; CI, confidence interval; ED, emergency room; GP, general practitioner; PR, prevalence ratio

‡ In case of GP visits, other outpatient physician or ED visits, and hospitalizations, the p-value refers to a p for trend

† Except malignant neoplasm of skin

* Health care contacts refer to hospitalizations within 12 months, or GP visits, ED or outpatient clinic/specialist visits, receipt of informal care, any nursing home visits, or permanent nursing home residency within 6 months prior to the baseline visit

†† defined as care received by relatives or other close persons

§§ Questionnaire-based health status on a 1 to 0 scale. A value of 1 corresponds to perfect health and a value of 0 to death

** The CCI predicts 10-year survival in patients with multiple comorbidities and ranges from 0 to 33 points. Lower scores indicate a higher risk 10-year-survival. 7 points correspond to an estimated 0% 10-year survival
