# Supplementary material for: Cross-sectional study on the prevalence of influenza and pneumococcal vaccination and its association with health conditions and risk factors among hospitalized multimorbid older patients
Source: PLoS One. 2021 Nov 16;16(11):e0260112. doi: 10.1371/journal.pone.0260112 (PMC8594840; doi:10.1371/journal.pone.0260112)
Supplement: S2 Table — (DOCX) [file pone.0260112.s002.docx]

|  | **Adjusted PR**§ | **95 % CI** | **p-value**‡ | **Adjusted PR**§ | **95% CI** | **p-value**‡ |
| --- | --- | --- | --- | --- | --- | --- |
| **Influenza Vaccination Pneumococcal Vaccination** | | | | | | |
| **Clinical risk groups** |  |  |  |  |  |  |
| Chronic heart disease | 1.01 | 0.98-1.03 | 0.67 | 0.81 | 0.63-1.03 | 0.09 |
| Chronic respiratory disease | 1.21 | 1.09-1.35 | <0.001 | 2.06 | 1.27-3.37 | 0.004 |
| Chronic liver disease | 0.94 | 0.78-1.12 | 0.49 | 0.99 | 0.63-1.54 | 0.96 |
| Chronic kidney disease | 1.14 | 1.07-1.21 | <0.001 | 1.08 | 0.80-1.45 | 0.62 |
| Diabetes mellitus | 1.05 | 1.04-1.06 | <0.001 | 1.24 | 1.14-1.36 | <0.001 |
| Rheumatic disease | 1.01 | 0.95-1.07 | 0.78 | 1.11 | 0.83-1.47 | 0.49 |
| Any malignancy † | 1.03 | 0.99-1.08 | 0.11 | 1.18 | 0.93-1.50 | 0.17 |
| Immunosuppression | 0.98 | 0.84-1.13 | 0.74 | 1.27 | 0.98-1.66 | 0.08 |
| **Health care contacts*** |  |  |  |  |  |  |
| GP visits, n 0 | *Reference* | |  | *Reference* | |  |
| 1-2 | 1.11 | 0.89-1.37 | 0.11 | 2.32 | 1.59-3.40 | <0.001 |
| 3-4 | 1.12 | 0.89-1.31 |  | 2.92 | 2.74-3.11 |  |
| ≥ 5 | 1.23 | 0.93-1.61 |  | 3.44 | 2.70-4.37 |  |
| Other outpatient physician or ED visits, n 0 | *Reference* | |  | *Reference* | |  |
| 1-2 | 1.13 | 1.04-1.22 | 0.013 | 1.07 | 0.64-1.78 | 0.46 |
| ≥ 3 | 1.14 | 1.03-1.26 |  | 1.42 | 0.55-3.66 |  |
| Hospitalizations, n 0 | *Reference* | |  | *Reference* | |  |
| 1 | 1.04 | 0.97-1.11 | 0.81 | 0.92 | 0.82-1.04 | 0.77 |
| ≥ 2 | 0.99 | 0.92-1.07 |  | 1.03 | 0.82-1.30 |  |
| Nursing home resident | 1.00 | 0.89-1.13 | 0.96 | 0.99 | 0.65-1.50 | 0.96 |
| Any home nursing visits | 1.00 | 0.96-1.05 | 0.84 | 1.14 | 0.89-1.45 | 0.31 |
| Receipt of informal care†† | 1.12 | 0.97-1.38 | 0.12 | 1.33 | 0.74-2.41 | 0.34 |
| **Health scores** |  |  |  |  |  |  |
| EQ-5D < mean§§ | 1.03 | 0.99-1.07 | 0.13 | 1.28 | 0.72-2.26 | 0.41 |
| CCI ≥ 7****** | 1.10 | 1.03-1.19 | 0.007 | 1.06 | 0.93-1.21 | 0.41 |

Abbreviations: CCI, Charlson comorbidity index; CI, confidence interval; ED, emergency room; GP, general practitioner; PR, prevalence ratio; VAS, visual analog scale

§ adjusted for age, sex, ethnicity, education, alcohol consumption and smoking status

**‡** In case of GP visits, other outpatient physician or ED visits, and hospitalizations, the p-value refers to a p for trend

† Except malignant neoplasm of skin

***** Health care contacts refer to hospitalizations within 12 months, or GP visits, ED or outpatient clinic/specialist visits, receipt of informal care, any nursing home visits, or permanent nursing home residency within 6 months prior to the baseline visit

†† defined as care received by relatives or other close persons

§§ Questionnaire-based health status on a 1 to 0 scale. A value of 1 corresponds to perfect health and a value of 0 to death

****** The CCI predicts 10-year survival in patients with multiple comorbidities and ranges from 0 to 33 points. Lower scores indicate a higher risk 10-year-survival. 7 points correspond to an estimated 0% 10-year survival
