# Supplementary material for: Cross-sectional study on the prevalence of influenza and pneumococcal vaccination and its association with health conditions and risk factors among hospitalized multimorbid older patients
Source: PLoS One. 2021 Nov 16;16(11):e0260112. doi: 10.1371/journal.pone.0260112 (PMC8594840; doi:10.1371/journal.pone.0260112)
Supplement: S3 Table — (DOCX) [file pone.0260112.s003.docx]

|  | **Unadju-sted PR** | **95 % CI** | **p-value**‡ | **Adjusted PR**§ | | **95% CI** | **p-value**‡ |
| --- | --- | --- | --- | --- | --- | --- | --- |
| **Clinical risk groups** |  |  |  | |  |  |  |
| Chronic heart disease | 1.07 | 0.97-1.18 | 0.16 | | 1.09 | 0.88-1.36 | 0.44 |
| Chronic respiratory disease | 1.56 | 1.35-1.80 | <0.001 | | 1.49 | 1.37-1.62 | <0.001 |
| Chronic liver disease | 0.87 | 0.72-1.05 | 0.15 | | 1.04 | 1.01-1.06 | 0.014 |
| Chronic kidney disease | 1.11 | 0.98-1.26 | 0.11 | | 1.10 | 0.85-1.42 | 0.48 |
| Diabetes mellitus | 1.21 | 1.00-1.47 | 0.05 | | 1.23 | 1.07-1.42 | 0.004 |
| Rheumatic disease | 1.18 | 0.67-2.01 | 0.56 | | 1.03 | 0.67-1.60 | 0.89 |
| Any malignancy† | 1.25 | 1.03-1.52 | 0.027 | | 1.31 | 0.93-1.84 | 0.12 |
| Immunosuppression | 1.38 | 1.33-1.43 | <0.001 | | 1.28 | 1.21-1.35 | <0.001 |
| **Health care contacts*** |  |  |  | |  |  |  |
| GP visits, n 0 | *Reference* | |  | | *Reference* | |  |
| 1-2 | 2.11 | 1.71-2.60 | <0.001 | | 1.97 | 1.39-2.81 | <0.001 |
| 3-4 | 2.35 | 2.00-2.75 |  | | 2.32 | 2.30-2.33 |  |
| ≥ 5 | 2.66 | 2.51-2.82 |  | | 2.44 | 1.70-3.50 |  |
| Other outpatient physician or ED visits, n 0 | *Reference* | |  | | *Reference* | |  |
| 1-2 | 0.77 | 0.66-0.90 | <0.001 | | 0.83 | 0.78-0.88 | 0.21 |
| ≥ 3 | 0.74 | 0.71-0.77 |  | | 0.85 | 0.66-1.10 |  |
| Hospitalizations, n 0 | *Reference* | |  | | *Reference* | |  |
| 1 | 0.88 | 0.82-0.95 | 0.86 | | 0.87 | 0.81-0.93 | 0.82 |
| ≥ 2 | 1.02 | 0.81-1.29 |  | | 0.97 | 0.75-1.25 |  |
| Nursing home resident | 1.22 | 0.91-1.64 | 0.18 | | 1.12 | 0.50-2.51 | 0.78 |
| Any home nursing visits | 0.99 | 0.91-1.08 | 0.85 | | 0.96 | 0.90-1.02 | 0.16 |
| Receipt of informal care†† | 0.97 | 0.77-1.23 | 0.82 | | 0.86 | 0.76-0.96 | 0.011 |
| **Health scores** |  |  |  | |  |  |  |
| EQ-5D < mean§§ | 0.95 | 0.87-1.03 | 0.23 | | 0.98 | 0.96-1.00 | 0.06 |
| CCI ≥ 7****** | 1.24 | 1.01-1.51 | 0.04 | | 1.25 | 1.15-1.37 | <0.001 |

Abbreviations: CCI, Charlson comorbidity index; CI, confidence interval; ED, emergency room; GP, general practitioner; PR, prevalence ratio

§ adjusted for age, sex, ethnicity, education, alcohol consumption and smoking status

**‡** In case of GP visits, other outpatient physician or ED visits, and hospitalizations, the p-value refers to a p for trend

† Except malignant neoplasm of skin

***** Health care contacts refer to hospitalizations within 12 months, or GP visits, ED or outpatient clinic/specialist visits, receipt of informal care, any nursing home visits, or permanent nursing home residency within 6 months prior to the baseline visit

†† defined as care received by relatives or other close persons

§§ Questionnaire-based health status on a 1 to 0 scale. A value of 1 corresponds to perfect health and a value of 0 to death

****** The CCI predicts 10-year survival in patients with multiple comorbidities and ranges from 0 to 33 points. Lower scores indicate a higher risk 10-year-survival. 7 points correspond to an estimated 0% 10-year survival
